# QSAR analysis and molecular docking simulation of norepinephrine transporter (NET) inhibitors as anti-psychotic therapeutic agents

**DOI:** 10.1016/j.heliyon.2019.e02640

**Published:** 2019-10-19

**Authors:** Sabitu Babatunde Olasupo, Adamu Uzairu, Gideon Shallangwa, Sani Uba

**Affiliations:** a National Agency for Food and Drug Administration and Control (NAFDAC), Nigeria; b Department of Chemistry, Ahmadu Bello University Zaria, Nigeria

**Keywords:** Pharmaceutical chemistry, Genetic function approximation, Drug, Density functional theory, Norepinephrine transporter, Antipsychotic, QSAR

## Abstract

The norepinephrine transporter (NET) is a Na^+^/Cl^−^ coupled neurotransmitter transporter responsible for reuptake of released norepinephrine (NE) into nerve terminals in the brain, a key therapeutic used in the treatment of psychiatric disorders. A quantitative structural activity relationship (QSAR) study was performed on 50 compounds of NET inhibitors to investigate their inhibitory potencies against norepinephrine transporter as novel drugs for anti-psychotic disorders. The compounds were optimized by employing Density functional theory (DFT) with basis set of B_3_LYP/6-31G*. The genetic function Algorithm (GFA) approach was used to generate a highly predictive and statistically significant model with good correlation coefficient R^2^_Train_ = 0.952 Cross validated coefficient Q^2^_cv_ = 0.870 and adjusted squared correlation coefficient R^2^_adj_ = 0.898. The predictability and accuracy of the developed model was evaluated through external validation using test set compound, Y-randomization and applicability domain techniques. The results of Molecular docking analysis by using two neurotransmitter transporters PDB ID 2A65 (resolution = 1.65 Å) and PDB ID 4M48 (resolution = 2.955 Å) showed that two of the ligands (compound 12 and 44) having higher binding affinity were observed to inhibit the targets by forming hydrogen bonds and hydrophobic interactions with amino acids of the two receptors respectively. The results of these studies would provide important new insight into the molecular basis and structural requirements to design more potent and more specific therapeutic anti-psychotic drugs/agents.

## 1 Introduction

Psychotic disorder is a clinical syndrome of mental disorders in which some loss of contact with reality has occurred and it is generally applied to persons whose mental functioning is sufficiently impaired to interfere with their capacity to meet the ordinary demand of life [1]. Psychotic disorders are common to all countries and cause immense human suffering, social exclusion, disability, poor quality of life, staggering economic and social costs. It is estimated that one in every four people have a mental disorder [1]. The combined costs of mental disorder, including loss of productivity, loss of earning due to illness and social costs, are estimated to total at least USD 113 billion annually [2]. The major depressive disorders (MDDs) had been estimated as the second largest global burden among all diseases by 2030 which makes the discovery of novel and efficacious anti-psychotic drugs very urgent [3]. Persons with psychotic disorder are at risk for complications and derivatives effects of psychosis such as suicide attempts, substance abuse, homelessness, victimization by others and committing act of violence [4].

Norepinephrine (NE) is a neurotransmitter, a crucial neurochemical messenger employed in central noradrenergic and peripheral sympathetic synapses [5] responsible for reuptake of released norepinephrine (NE) into nerve terminals in the brain. Dysregulation of this neurotransmitter is associated with many debilitating Psychotic disorders and mental illnesses [6]. Inhibition of the norepinephrine transporter by NET inhibitors has emerged as important drug targets with a multitude of therapeutic potentials for the treatment of psychiatric disorders and mental diseases [7].

Quantitative structure-activity relationship (QSAR) analysis is a useful technique to find correlations between biological activities and molecular descriptors of different classes of compound [8]. QSAR plays a significant role in novel drug discovery, and it finds application in predicting the activity of novel compounds by mathematical expression which figure out the relationships between a chemical structure to their biological activity and a QSAR models give information that is very useful for drug design and medicinal chemistry.

In recent time, computer assisted drug design base on QSAR has been of great important to develop novel medications for the treatment of different ailments [9].

The aim of this study is to build up a QSAR model to explore the inhibitory potency of some NET inhibitors and to likewise elucidate the interactions between the inhibitor compounds, and the receptor site.

## 2 Materials and methods

### 2.1 Dataset collection and geometry optimization

A dataset of fifty (50) compounds of norepinephrine transporter (NET) inhibitors were sourced from CHEMBL Database.

Optimization is the process of finding the equilibrium or concept energy geometry of molecules. Chemdraw software ultra-version 12.0 was used to draw the chemical structures of the compounds and subsequently imported into Spartan 14 software [10] to optimize the molecular geometry at the Density Functional Theory (DFT) using the B_3_LYP at 6-31G* basis set [11] to generate quantum chemical and molecular descriptors.

### 2.2 Division of dataset

The dataset of the studied compounds was partitioned into a training set and a test set by using Kennard stone algorithm [12] “Dataset Division GUI 1.2” software. The training set was used to develop the QSAR model, while the test set was employed to validate the developed model.

### 2.3 Model building

A statistical analysis by genetic function approximation (GFA) techniques in the Material studio software 8.0 version was used to build the QSAR models. GFA has a distinctive attribute to generate a population of model equations rather than a singular model as most other statistical methods do. It also selects the basic function genetically, generate better models than those made using stepwise regression techniques. The range of variations in this population gives added information on the quality of fit and importance of the descriptors [13]. The Friedman's Lack of Fit (LOF) was employed to evaluate the quality of the model as a method that measures fitness of a model. LOF is estimated by this mathematical expression;

(1) $$LOF=\frac{SEE}{{\left(1-\left(C+d\times p\right)/M\right)}^{2}}$$

Where c is the number of basic functions, d is the smoothing parameter, M is the number of samples in the training set, SSE is the sum of square error and p is the sum number of descriptors contained in the model.

### 2.4 Molecular descriptors calculation

Molecular descriptors are arithmetical values that describe properties of molecules obtained from a well-defined algorithm or experimental procedure. The OD, ID, 2D and 3D molecular descriptors were calculated using paDel-Descriptor software 2.20 version [14] in addition to quantum chemical descriptors generated by the Spartan 14 software.

### 2.5 Data pre-treatment

Data pre-treatment for the generated molecular descriptors after normalization was done by using “Data pretreatment GUI 1.2” software that uses V-WSP algorithm [15] to remove noise and redundant data. This helps to overcome productivity and generalization failure of the model due to constant value and highly correlated descriptors in forming QSAR models.

### 2.6 Data normalization and descriptors transformation

Molecular descriptors values were normalized by employing " normalized data 1.0 version software " to give each variable the same opportunity and make the relationship between descriptors considerably less demanding. The molecular descriptors of the training set were transformed through normalization [16] using the mathematical equation below;

(2) $$x^{n}=\frac{X-X_{max}}{X_{max}-X_{min}}$$

Where X^n^ is the normalized descriptor, X_max_ is the maximum value in a descriptor column and X_min_ is the minimum value in the column of the training dataset.

### 2.7 Assessing quality assurance of the model

Statistical parameters of the model were reviewed and evaluated to ascertained its fitting ability, reliability, predictive ability, stability and robustness of the model generated. The quality assurance of a developed model is guaranteed if the following parameters are satisfied; R^2^ > 0.6, R^2^_pred_>0.5, Q^2^ > 0.6, *P* (95%) <0.05, high value of F-test, low values of R^2^_random_ and Q^2^_random_.

### 2.8 Validation of the model

Leave-one-out cross validation technique was employed to determine the predictive power of the model. This was evaluated by using this mathematical expression;

(3) $$Q_{cv}^{2}=1-\left[\frac{\sum {\left(Y_{pred}-Y_{exp}\right)}^{2}}{\sum {\left(Y_{exp}-{\bar{Y}}_{training}\right)}^{2}}\right]$$

Where Y_pred_, Y_exp_ and $\bar{Y}$_training_ symbolized the experimental, the predicted and mean values of experimental activity of training set compounds.

Also, the square of the correlation coefficient for the test set (R^2^_test_) was evaluate for the predictive capacity of the developed model as part of the external validation technique. The closer the value of R^2^_test_ value to 1.0 the better the model. The R^2^_test_ is evaluated by using this mathematical equation;

(4) $$R_{test}^{2}=1-\frac{\sum {\left(Y_{predtest}-Y_{test}\right)}^{2}}{\sum {\left(Y_{predtest}-Y_{training}\right)}^{2}}$$

Where Y_pred_ and Y_test_ are the predicted and experimental activity values of the test set compounds. $\bar{Y}$_training_ is the mean (average) activity value of the training set.

### 2.9 Y – randomization test

Y – randomization is an important external validation technique to ascertained that a developed QSAR model is strong and reliable and is not inferred by luck. Y-randomization test is performed on the training dataset. The low values of R^2^ and Q^2^ is an indication that the model is very robust and highly reliable, and the _C_R^2^_P_ value of the model must be greater than 0.5 to pass the Y-randomization test. The _C_R^2^_P_ value is calculated by using this this mathematical formular;

(5) $$cR_{p}^{2}=R\;\times {\left[R^{2}-{\left(R_{r}\right)}^{2}\right]}^{2}$$

Where

_C_R^2^_P_ = coefficient of determination for Y-Randomization
R = Coefficient of correlation for Y-Randomization
R_r_ = Average “R” of random models.

#### 2.9.1 Degree of contribution of selected descriptors

The level of contribution of each descriptor in the model is determined by calculating its standardized regression coefficients *bj* using this mathematical equation;

(6) $$b_{j}=\frac{s_{j}b_{j}}{SY}=J=1,\ldots \ldots ..d$$

*b*_*j*_ is the regression coefficient of descriptor *j. S*_*j*_ and *S*_*y*_ are the standard deviations for each descriptor and activity respectively.

The descriptor of higher absolute standardized coefficient implies a greater importance to the rest of molecular descriptors.

#### 2.9.2 Multi-co-linearity evaluation

Multi-co-linearity estimation among descriptors selected by GFA analysis is evaluated using variance inflation factor (VIF) by the mathematical expression below;

(7) $$VIF_{i}=\frac{1}{1-R_{ij}^{2}}$$

Where R^2^_ij_ is the correlation coefficient of the multiple regression between the descriptor i and the rest j descriptors in the developed model [17].

#### 2.9.3 Assessment of the applicability domain of the model

Evaluation of the applicability domain of a model is a significant step to confirm that the developed model is capable to make a reliable prediction within the chemical space for which it was developed [16]. To describe the applicability domain of the QSAR model, the leverage approach was employed.

Leverage of a given dataset hi, is defined by this mathematical expression;

(8) $${H_{i}=x_{i}\left(X^{T}X\right)}^{-1}X_{i}^{T}$$

Where *xi* the descriptor row is vector of the considered compound *i*, hi is the n x k descriptor matrix of the training set compound used to generate the model.

The warning leverage (h*) is the limit of normal values of x outliers and is expressed mathematically as;

(9) $$h^{*}=\frac{3\left(p+1\right)}{n}$$

Where n = number of training compounds and P is the number of predictor variables (descriptors) in the model.

If the leverages hi < h* for the test compounds, it considered to be reliably predicted by the developed model.

The relevance area of the model in terms of chemical space is visualized by the plot of standardized residuals againt leverage values (Williams plot).

### 2.10 Molecular docking simulation

The molecular interactions studies were carried out on Dell computer system, with processor properties of Intel ® Core i5-6100U CPU Dual@2.30GHz, 12 GB (RAM) between the ligands and two neurotransmitter transporters (targets); the Crystal structure of LEUTAA, a bacterial homolog of Na+/Cl--dependent neurotransmitter transporters and X-ray structure of dopamine transporter elucidates antidepressant mechanism as to elucidate which of the NET inhibitors will have the best binding affinity against any of these two receptors, because the current structural findings of human monoamine neurotransmitters transporters (MATs) is based on X-ray crystal structures of bacterial and invertebrate homologs [18].

#### 2.10.1 Making of ligand and target

**Fig. 1:**
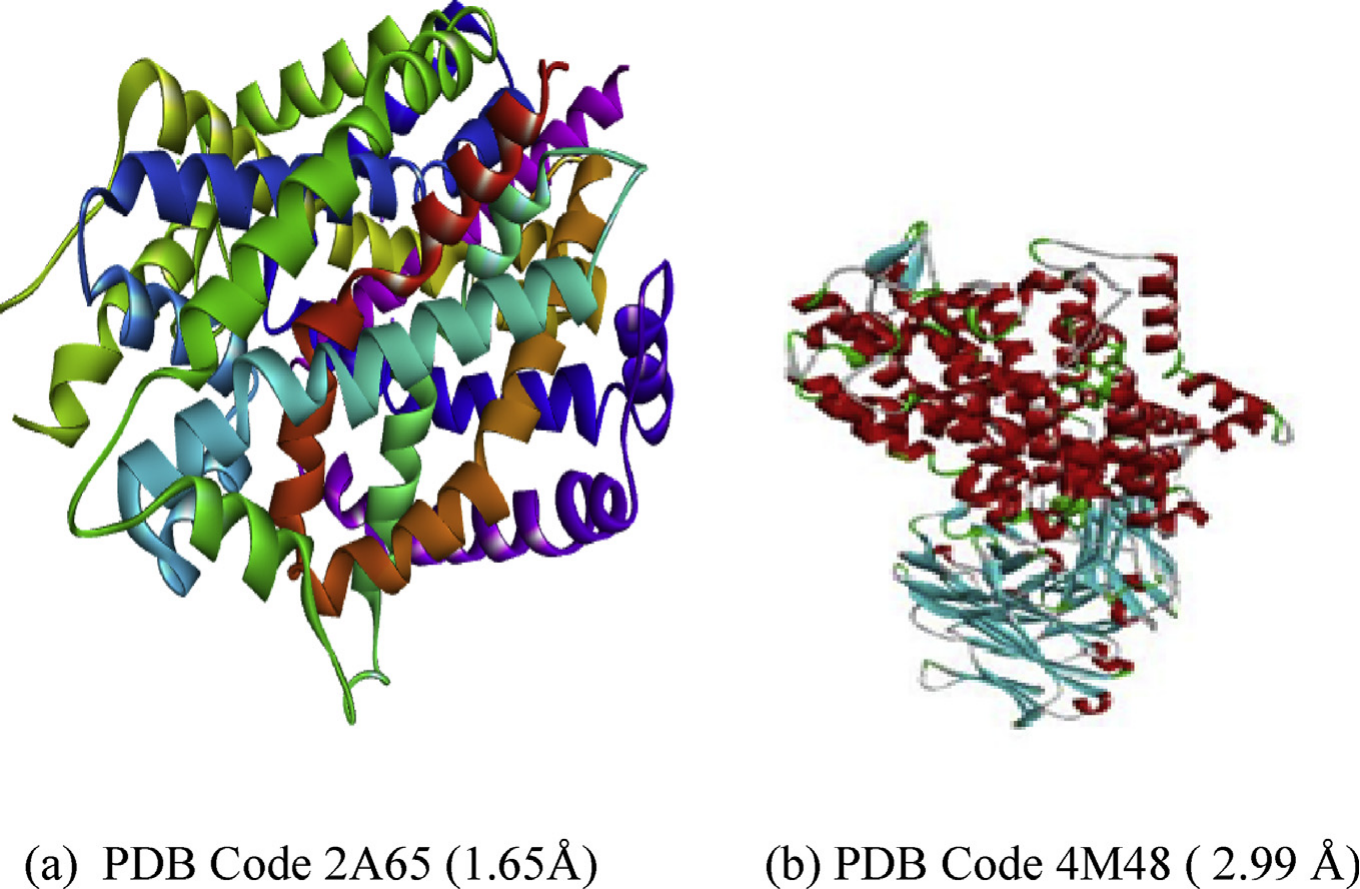
Prepared structures of the targets.

All the compounds were optimized using Spartan software initially saved as SDF files and were appropriately later saved as Protein Data Bank (PDB) files. Subsequently, Crystal structure of LEUTAA, a bacterial homolog of Na+/Cl--dependent neurotransmitter transporters and X-ray structure of dopamine transporter elucidates antidepressant mechanism (targets) were downloaded from Protein Data Bank website with PDB codes 2A65 and 4M48 respectively. Fig. 1 below displays the Prepared structure of the receptors.

#### 2.10.2 Docking process

The docking of the prepared ligands with the receptors 2A65 and 4M48 were conducted using the AutoDock Vina version 4.0 of Pyrex software. Hence, Discovery Studio software was used in visualizing the molecular interactions of the stable complex.

## 3 Results and discussion

QSAR study was explored to investigate the structure–activity relationship of 50 compounds with distinguishing organic fragments acting as norepinephrine transporter (NET) inhibitors. The nature of models in a QSAR study is expressed by its fitting the data points through regression and making predictions of isolated dataset.

### 3.1 QSAR on pKi of norepinephrine transporter (NET) inhibitors

A data set of 50 compounds was divided into a training set of 36 compounds used in developing the model and a test set of 14 compounds was used to evaluate the predictive ability of the QSAR model for the inhibition of norepinephrine transporter. The predicted and experimental activities alongside with their residual values were presented in Supplementary Table S1. The low residual values resulted from the experimental and predicted activities is a good indication that the developed model has good predictability.

The descriptive statistics parameters for the training set and test set activities value were reported in Table 1. By comparing the value of statistical parameters of the dataset activities in the Table; Mean (Training set = 6.940; Test set = 7.394), Variance (Training set = 1.227; Test set = 1.223), Standard deviation (Training set = 1.108; Test set = 1.106), Range (Training set = 4.439; Test set = 4.436) and Median (Training set = 7.054; Test set = 7.497), the values for the training set were approximately equal to that of test set. This shows that the test set is interpolative within the training set, and the similarity in the activity distribution of training set and test set. This is a good quality assurance that Kennard Stone's algorithm used in this research generates a test set that is a true reflection of the training set.

**Table 1:** Descriptive statistical analysis of NET inhibitor compounds.

Descriptive values	Training dataset	Test dataset
Dataset Number	36	14
Standard Error	0.185	0.296
Median	7.054	7.497
Standard Deviation	1.108	1.106
Sample Variance	1.227	1.223
Kurtosis	-0.632	2.677
Skewness	0.229	-1.264
Range	4.439	4.436
Minimum	5.084	4.500
Maximum	9.523	8.936
Mean	6.940	7.394

The genetic algorithm-multiple linear regression (GA-MLR) examination prompted the choice of 6 descriptors, which were eventually used to amassed a linear regression model for calculating pKi of norepinephrine transporter inhibitors within the chemical space of the model. The model with statistical significance was selected and represented by Eq. (10) below:

(10) pKI=2.788(ALogP)+3.382(AATS7i)+3.782(ATSC3p)+2.234(IC2)−5.147(GGI10)+3.728(RDF75u)+0.989

N_train_ = 36, R^2^_train_ = 0.9156, R^2^_adjusted_ = 0.8982, Q^2^_LOO_ = 0.8755, Outliers > 3.0 = 0 N_test_ = 14, R^2^_test_ = 0.5832

N is the total number of the datasets, R^2^ is the squared correlation coefficient, Q^2^_LOO_ is the squared cross-validation coefficients for leave one out. In the model, the number of ratio of training set data to the ratio number of descriptors present in the model was 6 and in agreement with Topliss ratio [19]. This implies that the developed model obeyed the QSAR semi-empirical rule of thumb [20]. The name and the symbol of the descriptors, the standardized regression coefficients (degree of contribution) and percentage contribution of the descriptors were reported in Table 2. The combined presence of 2D and 3D descriptors in the developed model is an evidence that these types of descriptors are able to characterize good antipsychotic activity of the compounds. The sign, magnitude and percentage contribution of each descriptor is not only to give critical information on the direction of influence of the descriptor but also pinpoint the strength of contribution to the activity of the compound.

**Table 2:** Names of the model descriptors and their respective degree of contribution.

Descriptor	Descriptor Name	Type	Degree of contribution	percentage of contribution
ALogP	Ghose-Crippen LogKow	2D	0.513	13.3
AATS7i	Average Broto-Moreau autocorrelation - lag 7/weighted by first ionization potential	2D	0.500	13.0
ATSC3p	Centered Broto-Moreau autocorrelation - lag 3/weighted by polarizabilities	2D	0.631	16.4
IC2	Information content index (neighborhood symmetry of 2-order)	2D	0.383	10.0
GGI10	Topological charge index of order 10	2D	-1.061	27.6
RDF75u	Radial distribution function - 075/unweighted	3D	0.756	19.7

The model generated was subjected to internal and external validations. The outcome of internal and external validations of the model is in conformity to Occam's razor rule. The generally acceptable QSAR Model Validation Tools and the validated parameters of the model were presented in Table 3. The values of validation parameters of the model were in agreement with generally acceptable QSAR Model Validation Tools reported in Table 3. This confirmed the reliability, stability and robustness of the developed model.

**Table 3:** Accepted QSAR model validation tools [21].

Validation Tools	Interpretation	Acceptable Value	Developed model Value	Remarks
R^2^	Co-efficient of determination	≥0.6	0.911	pass
P(95%)	Confidence interval at 95% confidence level	<0.05	2.446	pass
Q^2^cv	Cross-Validation Co-efficient	>0.5	0.870	pass
R^2^-Q^2^cv	Difference between R^2^ and Q	≤0.3	0.04	pass
N _Ext testset_	Minimum number of external and test sets	≥5	14	pass
R^2^_Testset_	Co-efficient of determination of external and test set	≥0.5	0.5850	pass
cR^2^_p_	Coefficient of determination for *Y*-randomization	>0.5	0.840	pass
R^2^_adj_	Adjusted R-squared	>0.6	0.893	Pass
VIF	Variance Inflation Factor	<10	1.4–4.4	Pass
t-test	t-Statistice value	>2	5–9	Pass

The Pearson's correlation matrix and other statistical tools employed for validation of the model were reported in Table 4. The low value in correlation coefficients between each pair of descriptors (<7.0) is a clear indication that there was no significant multi-collinearity among the descriptors in the developed model. The Variance Inflation Factor (VIF) values reported in the Table 4 were less than 10 and the t-statistics values were greater than 2 for all the descriptors. This is a quality assurance that the developed model was statistically significant, and the descriptors contributed appreciably to the model at 95% level [21] and they were orthogonal.

**Table 4:** Pearson's correlation matrix and model quality assurance.

	*ALogP*	*AATS7i*	*ATSC3p*	*IC2*	*GGI10*	*RDF75u*	VIF	t-statistics	p value
ALogP	1						1.5021	7.5604	2.47E08
AATS7i	-0.3321	1					1.4789	7.4649	3.16E-08
ATSC3p	-0.2592	-0.2991	1				1.4376	9.4970	2.1E-10
IC2	-0.2742	0.0487	0.0765	1			1.4177	5.8502	2.4E-06
GGI10	-0.2382	0.0921	-0.1711	0.5005	1		4.5022	-9.5663	1.79E-10
RDF75u	-0.2940	0.2215	-0.1337	0.4759	0.6377	1	4.3800	6.7912	1.87E-07

**Fig. 2:**
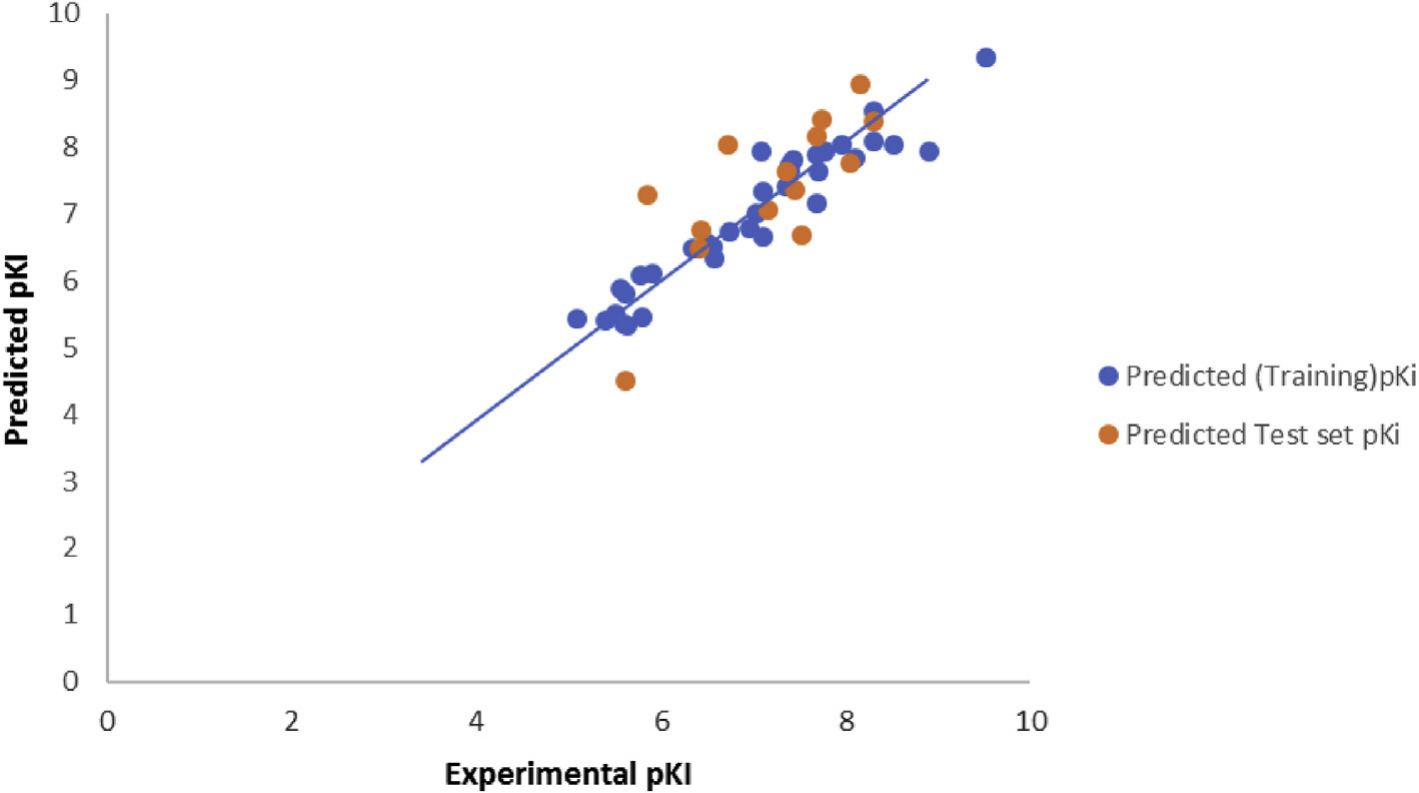
Plot of predicted pKi values against experimental pKi values for training and test sets.

**Fig. 3:**
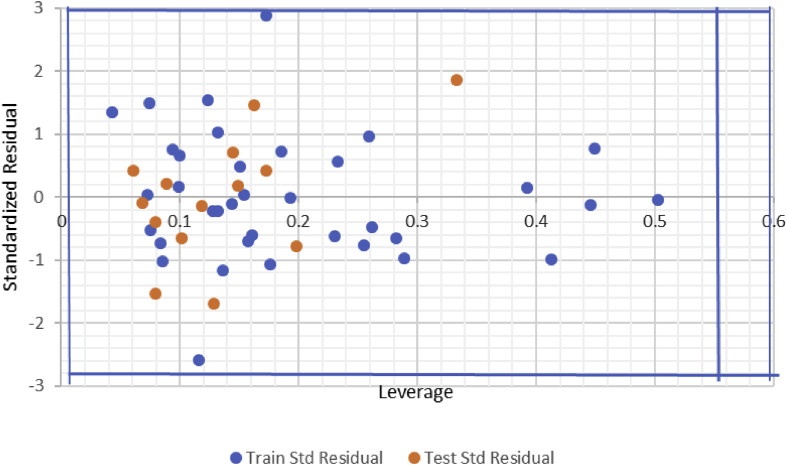
A Williams plot for the data set of pKi standardized residual against its descriptor space.

The model generated was used to predict the test set data, and the results were reported in Supplementary Table S1. The predicted pKi values for the training and test sets were plotted against the experimental pKi values as shown in Fig. 2, Similarly, the plot of the standardized residuals values for both the training and test sets against the leverage values of the descriptors in the model were shown in Fig. 3. As can be seen from Supplementary Table S1, Figs. 2 and 3, the calculated values for the pKi were in excellent agreement with those of the test set, as a result of this, no any form of error was displayed by the model.

### 3.2 QSAR model validation

The internal coherence of the training set was established by using leave-one-out cross-validation technique to ascertained the strength and reliability of the developed model, because the candid significance of a QSAR model is not merely their ability to mimic known activities of chemicals, set by their fitting power (R^2^), but above all is their prospective for guessing biological activity accurately. The great value of Q^2^_LOO_ for pKi of NET inhibitors used (0.8755) speak well of a fully clad internal validation of the model.

**Fig. 4:**
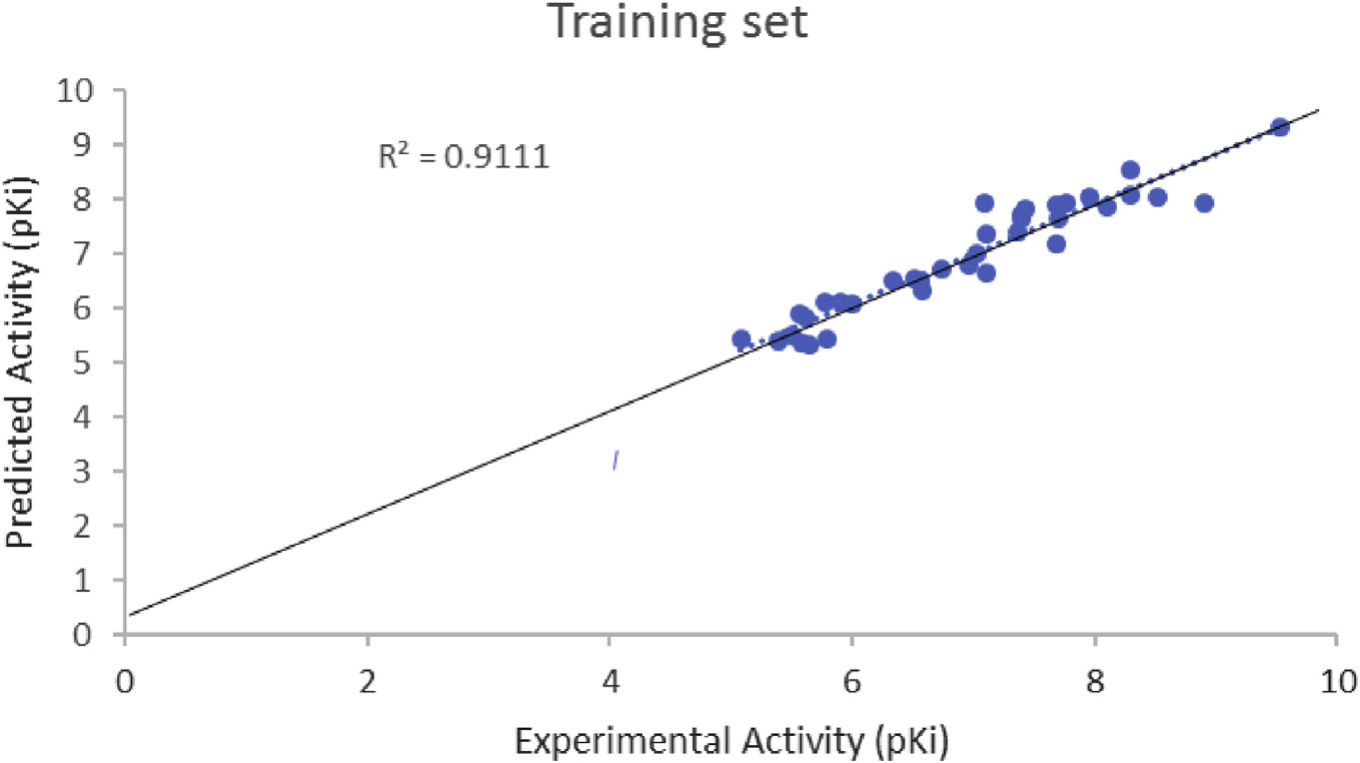
Plot of predicted pKi values against experimental pKi values for training.

The plot of experimental pKi values against predicted pKi values for training set was presented in Fig. 4. The displayed of linear relationship was observed in the plot between the experimental and predicted activities of the training set (R^2^ = 0.911). The fact that all these results were in agreement with QSAR validation tools presented in Table 3 is a confirmation of the reliability, robustness and stability of the developed model [21].

The Fig. 3, depicts the Williams plot of the NET dataset, in which the standardized residuals for each compound in the dataset were plotted against their leverage values, coming about to acknowledgment of likely outliers and outstanding chemicals in the models.

The applicability domain is set up inside a defined domain where all the data point were within the boundary ±3 for residuals and a leverage threshold h*($h*=3p^{o}/n$ where $p^{o}$ is the number of model parameters and n is the number of compounds) [15]. Based on our findings, it is clear that every one of the compounds of the training set and test set for the dataset were inside the domain (square area) and no statistical value far from others compounds (outlier) with standardized residuals >3*d* for the dataset exist.

The percentage of contribution was calculated to determine the relative importance and the contribution of every descriptor in the model. The degree of contribution of each descriptor and variance inflation factor (VIF) of the descriptor were estimated to evaluate the percentage and the significance of contribution of the descriptors as reported in Tables 2 and 4 respectively. The descriptor GGI10 showed highest contribution value (27.6%) in the model with VIF value of 4.502 as reported in the two tables, but the contribution negatively affects the model as it is observed in the Eq. (10) with negative regression coefficient.

The robustness and reliability of the model was evaluated through Y-randomization test to ascertain whether the developed model is by chance correlation or not. After few repeated trials to compare the stemmed scores with the scores of the original model with non-randomized data, the new QSAR model generated was observed to have low R^2^ and Q^2^_LOO_ values as reported in Table 5. The results of this test were clearly in agreement with QSAR validation tools presented in Table 3. This is an indication that the developed model is robust, good and dynamic. The fact that cR^2^_p_ value > 0.5, confirms that the model possesses good quality assurance and that the model is not only inferred by chance but also very powerful.

**Table 5:** Y-randomization table for QSAR Analysis.

Model	R	Rˆ2	Qˆ2
Original	0.9545	0.9111	0.8702
Random 1	0.4197	0.1762	-0.2759
Random 2	0.3402	0.1157	-0.4558
Random 3	0.3943	0.1555	-0.3333
Random 4	0.4690	0.2199	-0.2220
Random 5	0.4408	0.1943	-0.1861
Random 6	0.1560	0.0243	-0.6456
Random 7	0.3589	0.1288	-0.3166
Random 8	0.3237	0.1048	-0.3536
Random 9	0.3323	0.1104	-0.4357
Random 10	0.3646	0.1329	-0.3307
Random Models Parameters			
Average r:	0.3599		
Average rˆ2:	0.1363		
Average Qˆ2:	-0.3555		
cRpˆ2:	0.8439		

### 3.3 Elucidation of descriptors in NET pKi model

By interpreting the molecular descriptors presented in the model (Table 2), it is possible to increase supportive chemical functional groups, fingerprints and pharmacophores into the activities of the NET inhibitors. Therefore, a sufficient interpretation of the QSAR results is given below.

ALogP is a 2D type molecular descriptor, and the first in our QSAR model. It defined as Ghose-Crippen LogKow or Ghose-Crippen-Viswanathan octanol-water partition coefficient. (ALogP) is calculated from the AlogP model consisting of a regression equation based on the hydrophobicity contribution of 115 atom types [22, 23]. AlogP estimates are provided only for compounds having atoms of types C, H, O, N, S, Se, P, B, Si, and halogens.

Each atom in every structure is classified into one of the 115 atom types. Then, estimated logP for any compound is given by:

$$\text{A}\log\text{P}=\sum_{i}{\text{n}}_{i}{\text{a}}_{i}$$

where n is the number of atom of type *i* and a_*i*_ is the corresponding hydrophobicity constant. The list of the atom types with the corresponding hydrophobicity contributions is given under the list of atom-centered fragments. This descriptor tells us the higher the number of hetero atoms in a molecule, the higher the tendency for this molecule to be less hydrophobic. Since the percentage contribution of the descriptor in this model is 13%, it indicates that more than 10% of the bioactivity of a lead compound will improve should the number of heteroatoms present be increased.

AATS7i and ATSC3p are defined as Average Broto-Moreau autocorrelation - lag 7/weighted by first ionization potential and Centered Broto-Moreau autocorrelation - lag 3/weighted by polarizabilities respectively. They are both 2D autocorrelation descriptors and their respective percentage contribution to the models are given as 13 and 16.4% respectively in Table 1. The ATS descriptor describes how a property is distributed along the topological structure. It is a spatial autocorrelation on a molecular graph, which can be used to improve the activity of the compounds by altering the ionization potential and polarizability of the compounds. Since these molecular descriptors contributed positively to the model the pKi values of the compounds can be improved by adding fragments to the compounds that can increase the polarity of the compounds thereby creating the charge stability of the ligands' interaction with the binding sites. GGI10 is a topological charge descriptor defined as Topological charge index of order 10. GGI10 gave the highest contribution in the model, but since its contribution negatively affect the model, then the steady reduction in this descriptor value can improve the Ki values of the dataset. The ability of topological charge indices to describe molecular charge distribution has been established by correlating them with the dipole moment of a heterogeneous set of hydrocarbons, and so reducing the number of heterogeneous hydrocarbons presently correlated with the dipole moment of the molecule will lead to an increase in the bioactivity of the compounds.

IC2 is defined in Table 1 as Information content index (neighborhood symmetry of 2-order), it is a 2D type information content descriptor. It gave the least contribution to the model, but 10% contribution can be significant depending on the nature of the molecule. The IC2 molecular descriptor suggests that by introducing other bonds at that carbon, the structural complexity of the molecules will be increased and the Shannon entropy will also be increased thereby easily activating the interactions of the molecule with the binding site.

RDF75u is an RDF descriptor (Radial Distribution Function descriptors), this descriptor is based on the distance distribution in the geometrical representation of a molecule and constitute a radial distribution function code (RDF code) that shows certain characteristics in common with the 3D-MORSE code. The radial distribution function in this form meets all the requirements for a 3D descriptor, it also provides further valuable information such as bond distances, ring types, planar and non-planar systems. This fact is a most valuable consideration for a computer-assisted code elucidation [24]. The positive regression coefficient of this descriptor in the model as contained Eq. (7) with the highest value of degree of contribution as reported in Table 2 is a good indication of its influential contribution to the antipsychotic activity with variation in the bond distance and ring types of the studied compounds.

### 3.4 Docking result

The docking result of this study is presented in terms of binding affinity (kcal/mol) as reported in Supplementary Table S1. All the ligands were docked into the active site of the receptors, the Crystal structure of LEUTAA, a bacterial homolog of Na+/Cl--dependent neurotransmitter transporters and X-ray structure of dopamine transporter elucidates antidepressant mechanism in order to evaluate their abilities to inhibit these neurotransmitters. The current available findings of human neurotransmitters transporters are based on X-ray crystal structures of bacterial and invertebrate homologs which includes the bacterial amino acid transporters LeuT (PDB: 2A65) and the Drosophila melanogaster (PDB: 4M48) [18] as employed in this study.

**Fig. 5:**
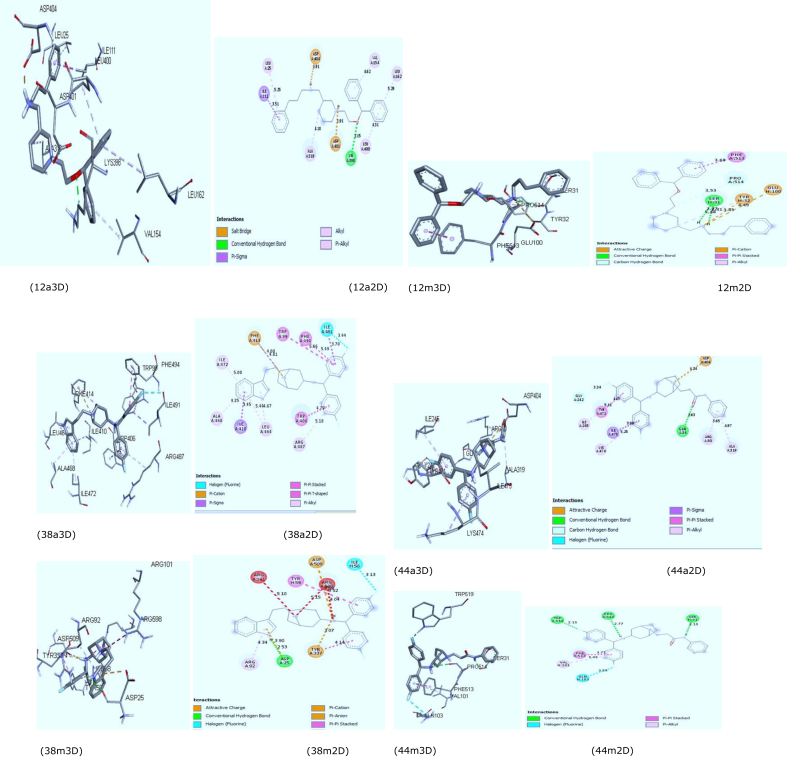
(12a 2D&3D), (38a 2D&3D) and (44a 2D&3D) depict 2D and 3D interactions at the binding site between receptor PDB code 2A65 with ligand 12, 38 and 44 while (12m 2D&3D), (38m 2D&3D) and (44m 2D&3D) show 2D and 3D interactions at the binding site between receptor PDB code 4M48 with ligand 12, 38 and 44 respectively.

The binding affinity values of the two receptors (PDB: 4M48 and PDB: 2A65) for all the studied compounds ranged from 4.4 kcal/mol to 10.3 kcal/mol and were reported in Supplementary Table S1. Ligands 8, 12,26, and 38 had higher binding affinity with the receptor PDB 4M48 and Ligands 9, 10, 12, 38 and 44 had higher binding affinity with the receptor PDB 2A65 respectively. The Discovery Studio Visualizer was used to visualize and analyze the three ligands of higher binding affinity that were found to display higher binding affinity and common to the two receptors as shown in Fig. 5.

The binding affinity, hydrogen bond, hydrophobic and electrostatic interactions of the three ligands having higher binding affinity with the two receptors were reported in Table 6. The number 12a, 38a & 44a represent the interactions of the Ligands (compound 12, 38 and 44) between the receptor (PDB ID 2A65) while 12m, 38m & 44m depict the interaction of the ligands (compound 12, 38 and 44) between the receptor (PDB ID 4M48) respectively.

**Table 6:** Molecular interactions between the three ligands of higher binding affinity and the two receptors.

Ligand CHEMBL ID	Ligand Number	Binding Affinity (kcal/mol)	Hydrogen bond		Hydrophobic interactions	Electrostatics Interactions
			Amino acid	Bond length (Å)	Amino Acid	Amino Acid
CHEMBL67078	12a	-9.3	LYS398	2.15279	ILE111, ALA319, VAL154, LEU162, LEU400, LEU25	
	12m	-7.35	SER31	2.76717	PHE513,TYR32	TYR32
			SER31	2.31044		
CHEMBL197384	38a	-10.3			ILE491, ILE410, TRP406,TRP99, PHE494,ARG487,LEU464.ALA464,ILE472	PHE414
	38m	-7.5	ASP25	2.53334	TYR337, TYR59, ARG92	ASP25
CHEMBL200310	44a	-9.9	GLN34	2.62533	ILE475, TYR471, ILE245, LYS474,ARG30,ALA319	ASP404
	44m	-8.45	PRO514	2.15327	PHE513, VAL101	
			SER31	2.76554		
			TRP519	2.1523		

All the three ligands (compound 12, 38 and 44) with the higher binding affinity were observed to inhibit the targets by forming hydrogen bonds and hydrophobic interactions with amino acids of the two receptors (PDB ID 2A65) and (PDB ID 4M48) respectively except compound 38 that could not form hydrogen bond with the receptor (PDB ID 2A65) as reported in Table 6. This may inform the higher resolution (2.99 Å) of the receptor (PDB ID 4M48) compare to the lower resolution (1.65Å) of the other receptor (PDB ID 2A65) (https://www. rcsb.org). The three ligands were found to be firmly bonded with Hydrogen bonds of the receptor (PDB ID 4M48) pocket amino acids (SER31, ASP25, PRO514 and TRP519). The higher number of Hydrogen bonds were observed in the two ligands (compound 12 and 44) with the target pockets of the receptor (PDB ID 4M48) which might be connected to their higher activity (compound 12, pKi = 7.383 and compound 44, pKi = 5.607) contrast with to the other ligand (compound 38, pKi = 5.084) with the lowest activity which formed just a single hydrogen bond with the receptor. This infers a direct relationship between the binding affinity and inhibitory activity of the studied compounds proved from the number of hydrogen bonds formed between the ligands and the receptor. However, high binding affinity is evident in the ligand 38 and this might be because of its large number of hydrophobic interactions and electrostatic effect due to the presence of fluorine atom, Pi- Cation, Pi- Sigma, Pi-Pi- stacked, Pi-Pi-T-shaped, Pi-Alkyl with amino acid residues (ILE491,ILE410, TRP406,TRP99, PHE494,ARG487,LEU464. ALA464,ILE472).

## 4 Conclusions

A QSAR investigation was performed on dataset of 50 norepinephrine transporter (NET) inhibitors, mined from CHEMBL database. The result of the QSAR modelling was reliable as it satisfies the OECD criteria for model development. The combination of 2D and 3D descriptors generate a good model to predict the inhibitory activity of the studied compounds. The internal validation was reported in the work to have a Q_2_cv = 0.870, while the external validation reported in R^2^_Pred_ value was given as 0.583, this implies a good predictive ability of the model.

The result of Applicability Domain (AD) shows that all the studied compounds were within the defined domain. Molecular docking study were carried out on all the compounds using two neurotransmitter transporters (receptors) PDB IDs 2A65 and 4M48 respectively. Three ligands (compound 12,38 and 44) showed higher binding affinity were found to best inhibit the two receptors by forming strong hydrogen bonds and hydrophobic interactions with amino acids of the targets. However, higher number of hydrogen bonds were observed between the receptor (PDB ID 4M48) and two ligands (compound 12 and 44) out of the three ligands with higher activity, compound 12 (pKi = 7.387) and compound 44 (pKi = 5.607) compare to compound 38 with the lowest activity (pKi = 5.084). This suggests excellent correlation between the binding affinity and inhibitory activity of the ligands and that the mechanisms or mode of action of the ligands could be a direct interaction with the receptor (PDB ID 4M48) of higher resolution (2.99 Å) value. Therefore, the two Ligands, compound 12 (1-(2-(benzhydryloxy)ethyl)-3-(((3-phenylpropyl)ammonio)methyl)piperidin-1-ium) and compound 44 (3-((bis(4-fluorophenyl)methyl)ammonio)-8-(3-oxo-3-(phenylamino)propyl)-8-azabicyclo[3.2.1]octan-8-ium) and the receptor PDB ID 4M48 (2.99 Å) proved to be the most promising hit compounds, and a good receptor for this study.

The information derived from the QSAR investigation and molecular docking analysis of this study could find a robust application in pharmaceutical industries to design novel NET inhibitors with more potent and more specific therapeutic anti-psychotic drugs.

## Declarations

### Author contribution statement

Sabitu Babatunde Olasupo, Sani Uba: Analyzed and interpreted the data; Wrote the paper.

Adamu Uzairu, Gideon Shallangwa: Analyzed and interpreted the data.

### Funding statement

This research did not receive any specific grant from funding agencies in the public, commercial, or not-for-profit sectors.

### Competing interest statement

The authors declare no conflict of interest.

### Additional information

No additional information is available for this paper.

## Appendix A Supplementary data

Suplementary Table S1_V2 Suplementary Table S1_V2
