# Supplementary material for: QSAR analysis and molecular docking simulation of norepinephrine transporter (NET) inhibitors as anti-psychotic therapeutic agents
Source: Heliyon. 2019 Oct 19;5(10):e02640. doi: 10.1016/j.heliyon.2019.e02640 (PMC6806411; doi:10.1016/j.heliyon.2019.e02640)
Supplement: Suplementary Table S1_V2 [file mmc1.doc]

**Table S1: The names of the dataset along with its Predicted, Experimental pKi and Binding affinity values**

| S/N | CHEM ID | NAME | Experimental pKi | Predicted pKi | Residual | Binding Affinity (kcal/mol)  PDB: 4M48 | Binding Affinity (kcal/mol)  PDB: 2A65 |
| --- | --- | --- | --- | --- | --- | --- | --- |
|  | CHEMBL32540 | (Z)-5-((Z)-2-(7a-methyl-1-(6-methylheptan-2-yl)hexahydro-1H-inden-4(2H)-ylidene)ethylidene)-4-methylenecyclohexane-1,3-diol | 6.000 | 6.072 | -0.073 | -7.2 | -8.6 |
|  | CHEMBL781 | 5-(4-chlorophenyl)-3,5-dihydro-2H-imidazo[2,1-a]isoindol-5-ol | 8.899 | 7.945 | 0.954 | -6.9 | -7.2 |
|  | CHEMBL808 | 1-(2-((4-chlorobenzyl) oxy)-2-(2,4-dichlorophenyl)ethyl)-1H-imidazole | 5.617 | 5.817 | -0.200 | -6.15 | -7.5 |
|  | CHEMBL822 | (E)-N,6,6-trimethyl-N-(naphthalen-1-ylmethyl)hept-2-en-4-yn-1-amine | 5.388 | 5.404 | -0.016 | -6.7 | -8.0 |
|  | CHEMBL828 | 10H-phenothiazine | 6.340 | 6.496 | -0.156 | -5.7 | -7.1 |
|  | CHEMBL42553 | methyl 3-(3,4-dichlorophenyl)-8-methyl-8-azabicyclo[3.2.1]octane-2-carboxylate | 8.301 | 8.531 | -0.230 | -6.15 | -6.9 |
|  | CHEMBL926 | N-(3,4-dihydroxyphenethyl)-4-(4-hydroxyphenyl)butan-2-aminium | 6.420 | 6.482 | -0.062 | -6.8 | -6.5 |
|  | CHEMBL482903 | 2-(3-(5-fluoro-1H-indol-3-yl)propyl)-6-methoxy-1,2,3,4-tetrahydroisoquinolin-2-ium | 5.775 | 6.095 | 0.010 | -7.4 | -8.0 |
|  | CHEMBL63703 | 4-((2-(benzhydryloxy)ethyl)ammonio)-1-benzylpiperidin-1-ium | 6.745 | 6.735 | 0.447 | -7.1 | -9.2 |
|  | CHEMBL67024 | 1-(2-(benzhydryloxy)ethyl)-4-((4-fluorophenethyl)ammonio)piperidin-1-ium | 7.102 | 6.655 | 0.316 | -7.0 | -8.9 |
|  | CHEMBL165 | (E)-5-(4-hydroxystyryl)benzene-1,3-diol | 5.639 | 5.323 | -0.252 | -6.25 | -7.1 |
|  | CHEMBL67078 | 1-(2-(benzhydryloxy)ethyl)-3-(((3-phenylpropyl)ammonio)methyl)piperidin-1-ium | 7.387 | 7.639 | -0.214 | -7.35 | -9.3 |
|  | CHEMBL99653 | 1-(1-hydroxycyclohexyl)-N,N-dimethyl-1-(3-(trifluoromethyl)phenyl)methanaminium | 7.678 | 7.891 | 0.047 | -6.5 | -6.7 |
|  | CHEMBL81 | 1-(2-(4-(6-hydroxy-2-(4-hydroxyphenyl)benzo[b]thiophene-3-carbonyl)phenoxy)ethyl)piperidin-1-ium | 6.553 | 6.506 | 0.159 | -8.0 | -8.5 |
|  | CHEMBL109571 | N,N-dimethyl-1-(3-(naphthalen-2-yl)bicyclo[2.2.1]heptan-2-yl)methanaminium | 6.955 | 6.796 | -0.854 | -7.0 | -8.3 |
|  | CHEMBL121027 | N1-(2-(benzhydryloxy)ethyl)-N2-(4-fluorophenethyl)-N1,N2-dimethylethane-1,2-diaminium | 6.432 | 6.751 | -0.319 | -6.0 | -7.9 |
|  | CHEMBL121611 | N1-(2-(bis(4-fluorophenyl)methoxy)ethyl)-N3-phenethylpropane-1,3-diaminium | 7.086 | 7.939 | 0.239 | -6.5 | -8.9 |
|  | CHEMBL123252 | N1-(2-(benzhydryloxy)ethyl)-N1,N3-dimethyl-N3-(3-phenylpropyl)propane-1,3-diaminium | 7.523 | 6.696 | 0.826 | -5.9 | -8.2 |
|  | CHEMBL121460 | N1,N2-bis(2-(bis(4-fluorophenyl)methoxy)ethyl)-N1,N2-dimethylethane-1,2-diaminium | 6.583 | 6.328 | -0.336 | -5.85 | -6.5 |
|  | CHEMBL1231 | 3-(2-cyclohexyl-2-hydroxy-2-phenylacetoxy)-N-isopropylprop-2-yn-1-aminium | 5.589 | 5.350 | 0.510 | -6.4 | -7.2 |
|  | CHEMBL139245 | 3-(4-chlorophenyl)-N-methyl-3-(naphthalen-1-yloxy) propan-1-aminium | 7.108 | 7.349 | 0.053 | -7.25 | -7.6 |
|  | CHEMBL139277 | N-methyl-3-(naphthalen-1-yloxy)-3-(m-tolyl)propan-1-aminium | 7.398 | 7.734 | -0.326 | -6.8 | -8.3 |
|  | CHEMBL141114 | N-methyl-3-(naphthalen-1-yloxy)-3-(p-tolyl)propan-1-aminium | 7.444 | 7.357 | 0.086 | -6.7 | -6.7 |
|  | CHEMBL142028 | N-methyl-3-(naphthalen-1-yloxy)-3-(thiophen-3-yl)propan-1-aminium | 7.678 | 7.168 | 0.186 | -6.25 | -7.4 |
|  | CHEMBL141681 | N-methyl-3-(naphthalen-1-yloxy)-3-phenylpropan-1-aminium | 7.699 | 7.645 | -0.383 | -6.55 | -6.5 |
|  | CHEMBL141974 | N-methyl-3-(naphthalen-1-yloxy)-3-(3-(trifluoromethyl)phenyl)propan-1-aminium | 7.155 | 7.074 | 0.081 | -7.35 | -8.0 |
|  | CHEMBL1289 | 1,2,4-trichloro-5-((3-iodoprop-2-yn-1-yl)oxy)benzene | 5.561 | 5.887 | -0.174 | -4.4 | -5.0 |
|  | CHEMBL153062 | 4-nonylphenol | 5.905 | 6.110 | 0.250 | -5.45 | -5.9 |
|  | CHEMBL179249 | 2-(phenyl(o-tolylthio)methyl)morpholin-4-ium | 9.523 | 9.337 | -0.355 | -6.75 | -7.1 |
|  | CHEMBL188248 | 1-(2-((2-aminophenyl)thio)phenyl)-N-methylmethanaminium | 7.426 | 7.809 | 0.492 | -5.4 | -6.5 |
|  | CHEMBL189374 | 1-(2-((2-amino-4-methylphenyl)thio)phenyl)-N-methylmethanaminium | 6.719 | 8.035 | -1.316 | -5.85 | -7.1 |
|  | CHEMBL190700 | 1-(2-((2-aminophenyl)thio)phenyl)-N,N-dimethylmethanaminium | 8.305 | 8.399 | -0.094 | -5.25 | -6.5 |
|  | CHEMBL194205 | N-methyl-3-(3-methyl-2-oxo-1-phenyl-1,2,3,4-tetrahydroquinolin-3-yl)propan-1-aminium | 7.357 | 7.636 | -0.279 | -5.9 | -7.4 |
|  | CHEMBL194781 | 3-(6-fluoro-2-oxo-1-(p-tolyl)-1,2,3,4-tetrahydroquinolin-3-yl)-N-methylpropan-1-aminium | 8.301 | 8.082 | -0.039 | 6.6 | -7.9 |
|  | CHEMBL196751 | 3-(3-ethyl-2-oxo-1-(p-tolyl)-1,2,3,4-tetrahydroquinolin-3-yl)-N-methylpropan-1-aminium | 7.769 | 7.942 | -0.072 | -6.3 | -6.7 |
|  | CHEMBL196110 | 3-(3-butyl-2-oxo-1-(p-tolyl)-1,2,3,4-tetrahydroquinolin-3-yl)-N-methylpropan-1-aminium | 8.097 | 7.846 | 0.010 | -5.75 | -6.9 |
|  | CHEMBL196368 | 3-(1-(3-fluorophenyl)-2-oxo-1,2,3,4-tetrahydroquinolin-3-yl)-N-methylpropan-1-aminium | 7.745 | 8.429 | -0.684 | -6.95 | -7.4 |
|  | CHEMBL197384 | 8-(2-(1H-indol-3-yl)ethyl)-3-((bis(4-fluorophenyl)methyl)ammonio)-8-azabicyclo[3.2.1]octan-8-ium | 5.084 | 5.438 | -0.319 | -7.5 | -10.3 |
|  | CHEMBL198033 | N-methyl-3-(2-oxo-1-(p-tolyl)-1,2,3,4-tetrahydroquinolin-3-yl)propan-1-aminium | 8.523 | 8.031 | 0.826 | -6.6 | -7.5 |
|  | CHEMBL198215 | 3-(6-chloro-3-methyl-2-oxo-1-(p-tolyl)-1,2,3,4-tetrahydroquinolin-3-yl)-N-methylpropan-1-aminium | 7.367 | 7.403 | 0.086 | -6.4 | -6.8 |
|  | CHEMBL197643 | 3-(1-(4-chlorophenyl)-2-oxo-1,2,3,4-tetrahydroquinolin-3-yl)-N-methylpropan-1-aminium | 7.678 | 8.177 | -0.499 | -6.7 | -6.8 |
|  | CHEMBL197707 | 3-((bis(4-fluorophenyl)methyl)ammonio)-8-(4-phenylbutyl)-8-azabicyclo[3.2.1]octan-8-ium | 5.788 | 5.449 | -1.316 | -7.0 | -7.9 |
|  | CHEMBL198807 | 3-(((4-chlorophenyl)(phenyl)methyl)ammonio)-8-methyl-8-azabicyclo[3.2.1]octan-8-ium | 5.499 | 5.504 | -0.094 | -7.35 | -7.2 |
|  | CHEMBL200310 | 3-((bis(4-fluorophenyl)methyl)ammonio)-8-(3-oxo-3-(phenylamino)propyl)-8-azabicyclo[3.2.1]octan-8-ium | 5.607 | 4.500 | 1.107 | -8.45 | -9.9 |
|  | CHEMBL198960 | N-methyl-3-(2-oxo-1-phenyl-4-propyl-1,2,3,4-tetrahydroquinolin-4-yl)propan-1-aminium | 8.155 | 8.936 | -0.781 | -6.25 | -7.3 |
|  | CHEMBL198842 | 3-(3,4-dichlorophenyl)-8-(2-(4-(9-(dimethyliminio)-11,11-dimethyl-3,4-dihydronaphtho[2,3-g]quinolin-1(2H,9H,11H)-yl)butanamido)ethyl)-2-(methoxycarbonyl)-8-azabicyclo[3.2.1]octan-8-ium | 6.511 | 6.550 | -0.499 | -7.5 | -8.4 |
|  | CHEMBL199116 | N-methyl-3-(2-oxo-3-propyl-1-(p-tolyl)-1,2,3,4-tetrahydroquinolin-3-yl)propan-1-aminium | 7.959 | 8.031 | 1.107 | -6.15 | -6.6 |
|  | CHEMBL198176 | N-methyl-3-(3-methyl-2-oxo-1-(p-tolyl)-1,2,3,4-tetrahydroquinolin-3-yl)propan-1-aminium | 8.046 | 7.760 | -0.781 | -6.4 | -7.6 |
|  | CHEMBL202410 | 1-(1-oxo-1-(p-tolyl)pent-4-en-2-yl)pyrrolidin-1-ium | 5.854 | 7.285 | 0.286 | -5.75 | -6.9 |
|  | CHEMBL201976 | 1-(1-(4-(furan-2-yl)phenyl)-1-oxopentan-2-yl)pyrrolidin-1-ium | 7.022 | 7.012 | -1.431 | -6.3 | -7.1 |
